# Reinforcing Nrf2 Signaling: Help in the Alzheimer’s Disease Context

**DOI:** 10.3390/ijms26031130

**Published:** 2025-01-28

**Authors:** Annamaria la Torre, Filomena Lo Vecchio, Valentina Soccorsa Angelillis, Carolina Gravina, Grazia D’Onofrio, Antonio Greco

**Affiliations:** 1 Laboratory of Gerontology and Geriatrics, Fondazione IRCCS Casa Sollievo Della Sofferenza, San Giovanni Rotondo, 71013 Foggia, Italy; f.lovecchio@operapadrepio.it (F.L.V.); cgravina@operapadrepio.it (C.G.); 2 Complex Unit of Geriatrics, Department of Medical Sciences, Fondazione IRCCS Casa Sollievo Della Sofferenza, San Giovanni Rotondo, 71013 Foggia, Italy; v.angelillis@operapadrepio.it (V.S.A.); a.greco@operapadrepio.it (A.G.); 3 Clinical Psychology Service, Health Department, Fondazione IRCCS Casa Sollievo Della Sofferenza, San Giovanni Rotondo, 71013 Foggia, Italy; g.donofrio@operapadrepio.it

**Keywords:** Alzheimer’s disease, oxidative stress, Nrf2, natural compounds, flavonoid compounds

## Abstract

Oxidative stress plays a role in various pathophysiological diseases, including neurogenerative diseases, such as Alzheimer′s disease (AD), which is the most prevalent neuro-pathology in the aging population. Oxidative stress has been reported to be one of the earliest pathological alterations in AD. Additionally, it was demonstrated that in older adults, there is a loss of free radical scavenging ability. The Nrf2 transcription factor is a key regulator in antioxidant defense systems, but, with aging, both the amount and the transcriptional activity of Nrf2 decrease. With the available treatments for AD being poorly effective, reinforcing the antioxidant defense systems via the Nrf2 pathway may be a way to prevent and treat AD. To highlight the predominant role of Nrf2 signaling in defending against oxidative stress and, therefore, against neurotoxicity, we present an overview of the natural compounds that exert their own neuroprotective roles through the activation of the Nrf2 pathway. This review is an opportunity to promote a holistic approach in the treatment of AD and to highlight the need to further refine the development of new potential Nrf2-targeting drugs.

## 1. Introduction

Alzheimer’s disease (AD) is a devastating neurodegenerative disorder for which there is currently no cure [1]. 

It is characterized by progressive memory loss, cognitive decay [2], and alteration in behavioral functions [1], creating the conditions for major vulnerability possibly leading to deadly complications. AD was officially listed as the seventh leading cause of death worldwide [3], with at least 55 million affected people worldwide. It has a massive impact on public health in terms of incidence, mortality, and morbidity but also in terms of cost of care and overall impact on family caregivers.

Considering direct costs (e.g., medical expenses, social services, and long-term care) and indirect costs (e.g., loss of productivity due to caregiving and premature mortality), in 2023, the total worldwide cost of dementia, including AD, was estimated to be around USD 1.3 trillion [4].

A solution to this scenario becomes even more urgent in view of the fact that, with dementia being an age-related pathology, the number of people affected is expected to double by 2050 [5] in the global aging population, with an increase in terms of cost of care expected to reach USD 2.8 trillion.

Pathogenetically, amyloid-β (Aβ) plaques and hyperphosphorylated tau tangles are the typical AD-related hallmarks, in addition to and in combination with neuroinflammation, which represents the third main hallmark of AD [6]. Neuroinflammation has been demonstrated to be closely linked to oxidative stress [7], which activates and exacerbates neurodegeneration in AD [8]. Free radicals, including ROS (Reactive Oxygen Species), are molecular species that contain an unpaired electron(s) in the outer atomic shell, which causes them to become mostly unstable. The ROS generation is very typical in biological systems, because they are produced by several metabolic processes of living organisms, including regulation and signaling, mitochondrial function, and cellular oxidation [9]. To provide protection against the potential damage of ROS, the human antioxidant system relies on endogenous and exogenous sources. While the endogenous antioxidant system is regulated by endogenous biomacromolecules, including glutathione peroxidase (GPx), glutathione (GSH), superoxide dismutase (SOD), and catalase (CAT), the exogenous antioxidant system includes molecules such as vitamins C and E, carotenoids, and polyphenols introduced by the diet [10].

With age, the endogenous antioxidant capacity progressively declines, causing an increase in oxidative species. As demonstrated in previous studies [11,12], the disequilibrium in the antioxidant activity is a primordial event in the AD brain. Additionally, the Aβ deposition itself contributes to cause the oxidative damage to DNA, proteins, and lipids in neurons, triggering microglial processes that are the major players in neuroinflammation [13].

Oxidative stress levels can determine cell death by altering physiological pathways, frequently through Ca^2+^ signaling. Indeed, oxidative stress promotes the influx of Ca^2+^ into the cytoplasm, from the extracellular compartment and from the sarcoplasmic and endoplasmic reticulum (SR/ER), through the cell membrane and specific channels. High cytoplasmic levels of Ca^2+^ promote the Ca^2+^ influx into nuclei and mitochondria, causing cell death [14]. In addition to apoptosis, the calcium dyshomeostasis in neurodegenerative diseases contributes to the deposition of Aβ, the hyperphosphorylation of the tau protein, and an abnormal synaptic plasticity, as well as making the formation of fibrillary structures by S100A9 more difficult [15]. The latter is a calcium-binding protein, which, with S100A8, protects cells from both protein phosphorylation and oxidative damage [16]. As confirmed in different studies on AD cases, in which S100A9-positive glial cells associated with widespread Aβ deposition were found, high levels of Ca^2+^ limit the peculiar role of S100A9 in modulating the neuroinflammatory response, favoring its association with the deposition of plaques of Aβ [17,18].

Additionally, ionic strength exerts a critical role in regulating oxidative stress and AD pathology by influencing ion channels [19], calcium signaling [20,21], enzyme [22] and mitochondrial function, neuroinflammation [19], and Aβ aggregation [23,24]. As demonstrated for Aβ aggregation, it was reported that ionic strength has a crucial involvement in the amyloid aggregation of alpha-synuclein (α-syn), to the point that ionic strength and protein concentration not only influence the structural variability in alpha-synuclein amyloid fibrils but also contribute to the formation of several types of aggregates [25].

To counteract oxidative stress in the brain, neurons, astrocytes, and microglia usually activate the endogenous antioxidant defense system, in which nuclear factor-erythroid-2-related factor 2 (Nrf2) represents the major mediator [26]. Indeed, Nrf2 is the master regulator of approximately 250 genes, having the Antioxidant Response Element (ARE) sequence in their promoter region. It has a role in several crucial molecular processes, including cellular metabolism, inflammatory modulation, and antioxidant response [27]. An increase in ROS beyond the threshold of equilibrium usually triggers the translocation of Nrf2 into the nucleus, where it binds to AREs, thus promoting the activation of an OS defense system [28].

It has been reported that the Nrf2 levels and their activation are reduced in the brain of older people [29].

Furthermore, in the AD brain, Nrf2 seems to be predominantly located in the cytoplasm while less remains in the nucleus, with a poor activation of AREs [30,31].

In addition, in APP/PS1 mice (a common mouse model of AD), it was observed that an overexpression of Nrf2 reinforces neuroprotection against Aβ toxicity [32].

## 2. Nrf2 Structure and Function

Together with Nrf1 and Nrf3, Nrf2 encoded by the NFE2L2 gene (Nuclear factor erythroid 2 like 2) is a member of the vertebrate cap’n’collar (CNC) transcription factor subfamily of basic leucine zipper (bZip) transcription factors [33].

**Figure 1:**
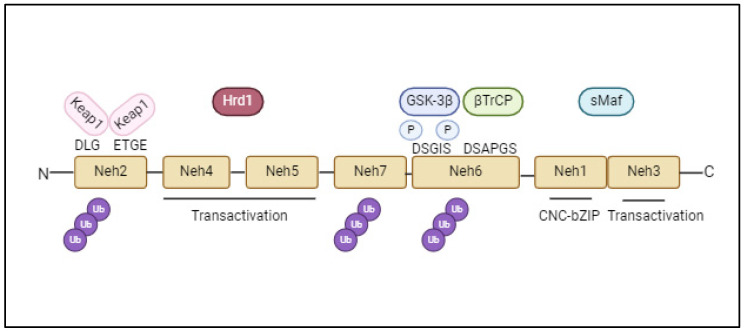
Domain architecture of the Nrf2 protein and molecular interactions.

It consists of 605 amino acids divided into seven highly conserved functional domains, referred to as Nrf2-ECH homology (Neh)—Neh1-7—which are not in order of sequence, for historical reasons (Figure 1). Meanwhile, Neh6 interacts with GSK-3β; Neh2 is an essential domain for regulating Nrf2 degradation mediated by a substrate adaptor protein, named Kelch-like ECH-associated protein 1 (Keap1), which is the chief regulator of Nrf2. In the cytosolic compartment, Keap1, as a dimer, usually functions as a substrate linker protein between the Cul3/Rbx1-based E3–ubiquitin ligase complex and Nrf2. This linker determines the continuous ubiquitination of Nrf2 and its consequent proteasomal degradation [34,35,36].

Upon oxidative stress, specific stress-sensing cysteine residues in Keap1 are modified, causing a change in conformational Keap1 structure that hampers the ubiquitination of Nrf2 by the Cul3 Rbx1-based E3–ubiquitin ligase complex [37]. In particular, the modification redoxes depending on cysteine sulfhydryl groups, especially those concerning the Cys151, Cys273, and Cys288 of Keap1, determining the dissociation of Nrf2 from Keap1.

The consequent stabilization of Nrf2 determines its nuclear translocation. Within the nucleus, Nrf2 heterodimerizes with the small Maf protein (sMaf) family (MafF, MafG, MafK) for the transcriptional activation of antioxidant genes [38].

The transcriptional activation reinforces the antioxidant defense systems, including enzymes such as glutathione peroxidase, catalase, and superoxide dismutase, and antioxidants such as glutathione, vitamin C, and vitamin E [39].

Regardless of Keap1, there are other mechanisms of Nrf2 regulation. Among them, there is the Nrf2/Bach1 signaling. Bach1 (broad complex, tramtrack and bric à brac and cap’n’collar homology 1) is a transcriptional repressor of Nrf2 and competes with Nrf2 for binding the same ARE sequence in several genes [40]. It was reported that with aging, Bach1 levels increase and, consequently, levels of Nrf2 reduce, leading to age-related changes in the expression of several gene-dependent Nrf2 [41].

Sensitive to oxidative stress, the protein kinase C (PKC) represents another Keap1-independent mechanism of Nrf2 regulation. In fact, in the presence of ROS, PKC, and also protein kinase CK2, it phosphorylates the Ser40 of Nrf2, promoting the dissociation of Nrf2 from Keap1 and their nuclear translocation [42]. A similar effect is also carried out by the phosphatidylinositide 3-kinases (PI3K)/Akt pathway [43], c-Jun N-terminal kinase (JNK), extracellular-regulated kinase (ERK) [44], and PERK (protein kinase R (PKR)-like endoplasmic reticulum kinase) [45].

Therefore, there is also an autophagic stabilization of Nrf2 that is mediated by the p62 protein, also named sequestosome 1 (SQSTM1), which is a ubiquitin-binding protein targeting aggregates for autophagic degradation. p62 acts as a competitor of Nrf2 for binding to Keap1. Indeed, p62 can directly interact with Keap1 for sequestering it into aggregates and promoting the translocation of Nrf2 into the nucleus. Here, the Nrf2 promotes the expression of many genes, including p62, with a positive loop of reinforcement [46].

**Figure 2:**
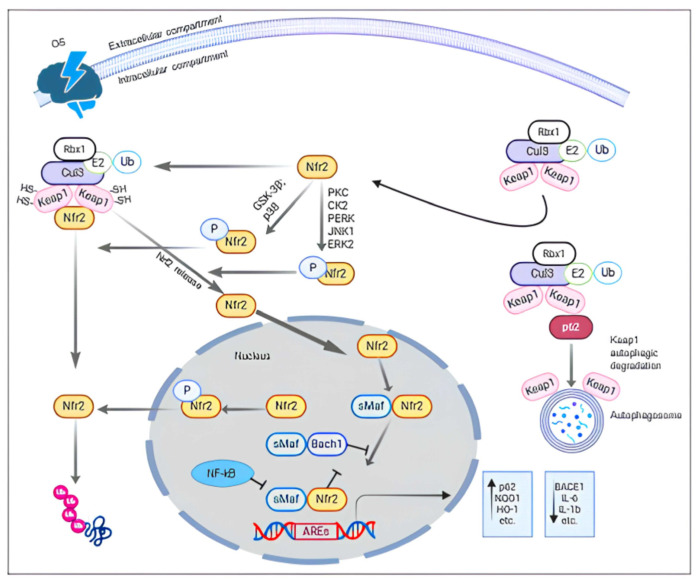
Physiological mechanisms of the Keap1-dependent and independent regulation of Nrf2. Oxidative stress leads to the positive regulation of the Nrf2 pathway by releasing Nrf2 from Keap1. The p62 increases Nrf2 translocation, triggering an autophagic degradation of Keap1. The Nrf2 phosphorylation mediated by CK2, PKC, PERK, ERK2, and JNK1 enhances the Nrf2 nuclear accumulation. In the nucleus, Nrf2 interacts with transcriptional co-activator Mafs to stabilize the ARE interaction for the transcriptional activation of antioxidant genes.

Instead, the glycogen synthase kinase 3-beta (GSK-3β), phosphorylating Nrf2, leads it to proteasomal degradation [47]. Beyond the Nrf2 modulation, GSK-3β is involved in several molecular mechanisms, including cell proliferation, apoptosis, and glycogen metabolism [48]. It was reported that GSK-3β is overexpressed in AD [49], reducing the Nrf2 activity in two different manners. Indeed, on one hand, GSK-3β, by phosphorylating Fyn, determines the phosphorylation of Nrf2 at Tyr568, causing the nuclear export of Nrf2, and its degradation [50]. On the other hand, the same GSK-3β is able to phosphorylate the Neh6 domain of Nrf2, promoting the binding of Nrf2- beta-transducin repeat-containing protein (β-TrCP) [46], preparatory to Nrf2 ubiquitination. Furthermore, GSK-3β is regulated by several kinases, including the above-mentioned kinase, such as the PI3K/Akt and p38-MAPK pathways. In the first case, GSK-3β is inactivated by the phosphorylation of its Ser9 PI3K/Akt mediation [51]. In the second case, the inactivation of GSK-3β depends on its phosphorylation at Thr390 p38-MAPK mediation (Figure 2) [52].

## 3. Nrf2 in the Brain: Physiological and Pathophysiological Conditions

Nrf2 exhibits a non-homogenous expression across the brain areas. Indeed, the expression level of Nrf2 is high in the *medulla oblongata*, which controls vital processes, and in the basal ganglia, involved in the control of emotions, executive functions, and movement [26]. At the same time, there is not a homogenous distribution of Nrf2 across the brain cells. Indeed, Nrf2 is highly expressed in microglia, astrocytes, and oligodendrocytes, and less in neurons [53].

Among brain cells, it was demonstrated that the astrocytes are actively involved in AD, because a substantial modification in the astrocyte function was observed in AD in in vitro and in vivo animal models as well as in AD patients’ brains [54].

Additionally, it has been observed that astrocytes contribute to the clearance and Aβ production by also expressing ApoE (apoliprotein E), which is frequently mutated in AD patients [55]. An accumulation of Aβ elicits a disequilibrium in astrocytic calcium signaling, causing a boost of ROS production [56].

The lower amount of Nrf2 in AD patients, refs. [57,58], causes an impairment of the cellular defense system against oxidative stress. This condition impacts the well-being of neurons, which usually have a low innate antioxidant defense system, to the point that they count on astrocytes for their protection. Additionally, an accumulation of Aβ determines the activation of the pathway of the nuclear factor kappa-light-chain-enhancer of activated B cells (NF-κB), causing the expression of astrocytic pro-inflammatory cytokines and chemokines with the promotion of neuroinflammation [59].

On the other hand, the deposition of Aβ increases tau phosphorylation and aggregation. This condition causes an additional increase in ROS levels, which contribute to oxidation products derived from lipids, nucleic acids, and proteins. While in physiological conditions, the presence of an oxidative environment favors Nrf2 activation, which, in turn, concurs to eliminate ROS and restore a redox equilibrium at a cellular level, in pathologic conditions, it is not possible to restore this balance. An impaired Nrf2 activity causes an additional imbalance between ROS production and cellular antioxidant defense response [60,61], with a progressive degeneration of brain cells.

**Figure 3:**
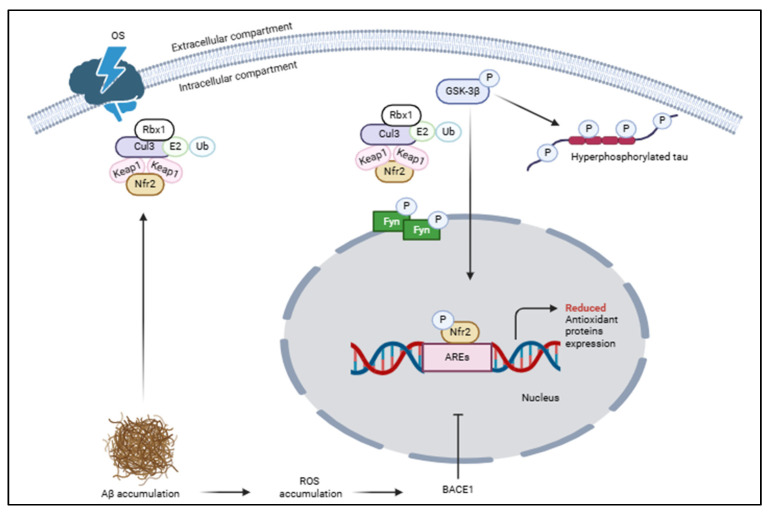
In AD, Aβ accumulation increases oxidative stress and blocks the Nrf2 activity by stabilizing the interaction between Keap1 and Nrf2, reducing the expression of antioxidant genes. Additionally, GSK-3β, involved in tau phosphorylation, favors Nrf2 degradation with its proteasomal degradation.

Despite, in AD, the mechanisms implying the reduced Nrf2 activity remaining unclear, an interesting link between the accumulation of Aβ and the reduced expression of Nrf2 was considered. Indeed, it was demonstrated that Aβ deposition enhances the levels of an Nrf2 suppressor, such as Keap1 and GSK-3β, with the consequent inhibition of Nrf2 signaling [62]. Other studies supposed that the same deposition of Aβ enhances the synthesis of Nrf2 and Keap1, as an initial protective response to the Aβ accumulation, but the continued upregulation of Keap1, also derived from oxidative stress, and an increased BACE1 activity, derived from the reduction in Nrf2 levels, turn on a vicious cycle leading to reduction in Nrf2 activity (Figure 3) [29,63].

Additionally, in AD patients’ brain, the dysfunction of autophagy was observed, in which Nrf2 is also implicated with a negative modulation [26].

Autophagy is a quality control system involved in the degradation of senescent organelles, proteins, and, in general, macromolecules, including misfolded proteins [64]. Indeed, autophagy is a key regulator for promoting the clearance of Aβ, and it is also the main pathway for removal of phosphorylated tau in neurons [65,66,67]. In autophagy, the activity of p62 is essential, because it binds polyubiquitinated proteins for presenting them to the autophagosome [68].

But an alteration both in the dysfunction of autophagy and in the inhibition of the Nrf2 pathway, typical of AD, determines a consequent accumulation of senescent organelles and misfolded proteins and an additional accumulation of ROS [69,70].

As described above, p62 represents an alternative way for Nrf2 modulation. It is plausible that from an impaired autophagy mechanism originates the p62 accumulation, responsible for the activation of Nrf2 signaling, but, considering reduced Nrf2 levels in AD, the consequence is an alteration of the clearance of Aβ and tau proteins, with their consequent accumulation. This loop enhances the autophagy impairment and neurodegeneration [26,71].

## 4. FDA-Approved Treatments for Alzheimer’s

To date, there are five FDA-approved drugs for AD clinical treatment. In particular, four of them are acetylcholinesterase inhibitors aimed at improving the cognitive functions and are tacrine, rivastigmine, galantamine, and donepezil. The other one, memantine, is an N-Methyl-D-aspartate (NMDA) receptor antagonist used for the treatment of moderate and severe AD. However, all of them may give only a transient improvement in cognitive ability and induce a wide range of side effects. Instead, a treatment based on molecules targeting Aβ and/or p-tau proved to be not very effective [72].

## 5. The Employment of Nrf2-Activating Natural and Synthetic Compounds for AD: The State of the Art

So far, the FDA has approved several clinical trials focused on reducing oxidative stress by modulating Nrf2 activity [73].

The multi-target therapy proposed a mix of antioxidants with AChE inhibitors or NMDA receptor antagonists, but, to reduce side effects, drug interactions, and costs, there are clinical trials studying several Nrf2-activating natural and synthetic compounds (Table 1).

While these compounds seem promising, there are potential shortcomings or limitations associated with their use because tests have not been conducted at a large scale to test the real efficacy and safety. Simultaneously, they should be engineered to develop better drug delivery systems, such as nanoparticles or liposomal formulations, especially to bypass the BBB and, in general, to improve the absorption by affected brain areas. Finally, employing advanced pharmacogenomics techniques should be useful for identifying patient subgroups that may be more or less susceptible to side effects.

## 6. Inside the Molecular Nrf2-Activating Mechanisms by the Action of Natural Compounds

A large number of epidemiological and experimental studies have remarked on the beneficial effects of some phenolic substances for preventing neurodegenerative diseases and other age-related pathologic conditions. Although the precise mechanisms remain obscure, many studies declare that these beneficial effects are shown through the ability to stimulate the defense gene system.

### 6.1. Rosmarinic Acid (ROSA)

**Figure 4:**
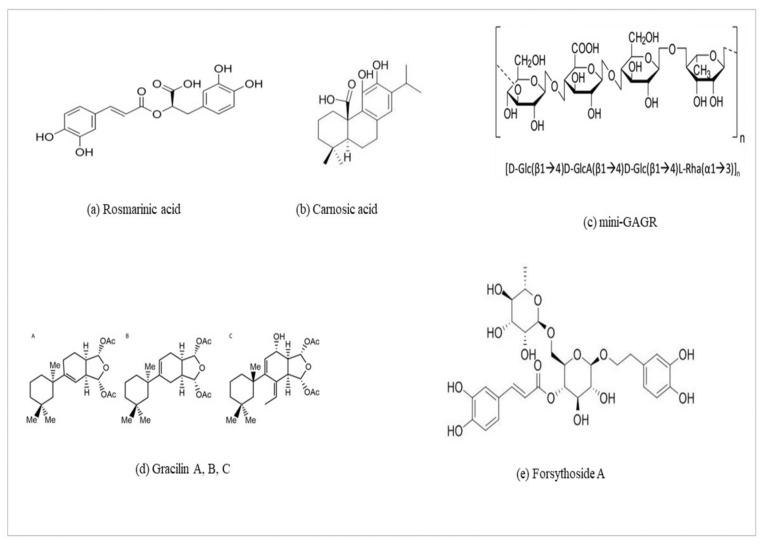
Chemical structure of natural compounds activating Nrf2 pathway.

Rosmarinic acid (Figure 4a) is a polyphenol with remarkable content present in the Lamiaceae including *Melissa officinalis*, commonly known as Lemon balm, and Rosmarinus [91].

This natural compound has many pharmacological functions including anti-inflammatory, antioxidant, anti-allergic, and antiviral activities [92,93]. It is likely that the protective role of ROSA is mainly exerted by the modulation of the PI3K/Akt pathway that, in turn, promotes the positive regulation of Nrf2 by inactivating GSK-3β.

Indeed, in two different in vitro studies, the ability of ROSA to promote the nuclear translocation of Nrf2 through the phosphorylation of Ser473 Akt, and GSK-3β inactivation, via the PI3K/Akt pathway was observed. The final outcome found by the authors was that the antioxidant response had been enhanced. Additionally, after administering ROSA, the authors, by Western blot and RT-qPCR experiments, registered high expression levels not only of Nrf2, but also of HO-1, SOD, and Bcl-2, and a concomitant reduced level of the Bax protein [94].

Given that the Nrf2 protein also manages apoptotic processes through the modulation of Bcl-2 and Bax levels [95,96], it is plausible that the activation of Nrf2 is the starting point for the modulation of both anti-apoptotic and antioxidant ROSA-dependent processes.

The protective role of ROSA is also explained by the modulation of Nrf2, exerted through the activity of mitogen-activated protein kinases (MAPKs), a family of highly related kinases including ERKs, JNKs, the p38 kinases, and other less known kinases. In a study carried out on human liver cell lines stressed with H_2_O_2_ exposition, Ding et al. demonstrated that the administration of ROSA ameliorates the oxidative stress of H_2_O_2_ induced by the activation of MAPK, and the consequent inactivation of GSK-3β, and Nrf2 expression. In general, while the phosphorylation of p38 and JNK promotes the apoptosis, the phosphorylation of ERKs has a protective role against oxidative stress. The authors demonstrated that the administration of ROSA ameliorates the oxidative damage by activating ERK expression and inhibiting JNK and p38. Overall, they revealed a diminished percentage of cell apoptosis, and an increased protein expression of NQO1, Nrf2, and MAPKs [97].

### 6.2. Carnosic Acid (CA)

Carnosic acid (CA) (Figure 4b) is a diterpenoid with remarkable content in the Lamiaceae, including *Rosmarinus* and *Salvia* [98].

The anti-inflammatory role of CA was specifically postulated, as explained below, in the Tamaki Y et al. work. Oxidative stress is responsible for the activation of CA, from the “pro-electrophilic state” to the “electrophilic state”, able to activate the Nrf2 pathway. Indeed, it was demonstrated that CA promotes the nuclear translocation of Nrf2 and its binding to AREs in the promoters of target phase 2 genes, to produce some enzymes with antioxidant and anti-inflammatory activities, among these being NAD(P)H quinone dehydrogenase 1 (NQO-1), heme oxygenase-1 (HO-1), GST (Glutathione S-Transferase), and γ-GCS [99].

It is likely that the antioxidant effect of CA is exerted by its binding to specific Keap1 cysteine residues, perturbing the interaction with Nrf2, thus stabilizing Nrf2. The protective role on brain CA-dependent cells has been observed by in vitro and in vivo studies.

In fact, in both in vivo and in vitro models of Parkinson’s disease (PD) and AD, an amelioration of neuronal and synaptic damage after treatment with CA via trans-nasal routing was registered. Histologically, in the hippocampus of treated transgenic mice models of AD, an increase in dendritic and synaptic markers and a decrease in the Aβ plaque number, astrogliosis, and tau tangles was observed [100]. Similarly, Wu CR et al. proved an improvement of behavioral activity and neuroprotective effects after treatment with CA in a rat model of PD previously exposed to 6-hydroxydopamine (6-OHDA). Additionally, by in vitro studies, the authors demonstrated that CA prevents the lipid 6-OHDA-dependent peroxidation, enhancing the expression of SOD1, γ-GCS, and glutathione reductase (GR) [101,102,103].

Studying the neuroprotective activity of CA in more detail, Chen JH et al. observed an attenuation of neurotoxin effects of 6-hydroxydopamine (6-OHDA) in SH-SY5Y cells. In particular, the authors demonstrated that the administration of CA was responsible for the downregulation of the pro-apoptotic JNK and p38 signaling pathways, mediated by the synthesis of GSH through an Nrf2-dependent mechanism [104]. Similarly, Miller DM et al. demonstrated the capacity of CA of mitigating the inhibition of mitochondrial respiration by 4-hydroxy-2-nonenal (4-HNE) in cortical mitochondria. The authors also linked the attenuation with the activation of the Nrf2 pathway [105].

Additionally, by in vitro studies, Kosaka K. et al. demonstrated [102] that CA enhances an increase in the nerve growth factor (NGF) production through an Nrf2-dependent pathway, with a consequent activation of Nrf2 target genes, including thioredoxin reductase 1 (TXNRD1) and heme oxygenase 1 (HO-1) [105].

Given the results and the ability to cross the blood–brain barrier (BBB), as a defender of neurons from oxidative stress and excitotoxicity, CA was proposed as a neuroprotective agent for the treatment of neurodegenerative diseases [106].

### 6.3. Mini-GAGR and Gracilins

Similarly to the CA, mini-GAGR (Figure 4c) also has the ability to cross the BBB; therefore, it could be a candidate for stimulating Nrf2 signaling and attenuating AD pathogenesis. It is a polysaccharide, produced by the cleavaging of low-acyl gellan gum [107]. According to a study carried out by Murphy et al., the intranasal administration of mini-GAGR in the 3xTg-AD mouse models is able to increase the activity of antioxidant enzymes that are Nrf2-dependent, decreasing the levels of ROS [11].

Treating mouse embryonic cortical neurons with mini-GAGR, the authors demonstrated that mini-GAGR is able to enhance the antioxidant defense system. Indeed, after the treatment, the authors observed high levels of the principal antioxidant enzymes. Additionally, after treatment with mini-GAGR, the authors observed a substantial increase in the Nrf2 transcriptional activity and an increased nuclear localization, proving that mini-GAGR is an Nrf2 activator [11]. Furthermore, after treatment with mini-GAGR and in the presence of pathological concentrations of H_2_O_2_ and 4-Hydroxynonenal (4HNE) [105], the authors demonstrated that the treatment with mini-GAGR is able to reduce ROS levels due to an increase in antioxidant enzymes [11].

Moreover, the authors clarified the mechanism by which mini-GAGR activates Nrf2. In particular, they demonstrated that mini-GAGR promotes the dissociation of Nrf2 from Keap1 in a PKC-dependent manner. Indeed, they registered an increase both in the enzymatic activity of PKC and of the Ser-40 phosphorylation of Nrf2 with a PKC-dependent manner [11]. Moreover, in an in vivo study, the authors demonstrated a multimodal effect of mini-GAGR in attenuating AD pathogenesis. In an AD animal model (3xTg-AD mice), the authors observed that to an intranasal administration of mini-GAGR corresponded an increased expression of antioxidant enzymes, including HO-1, SOD1, and GPx4 (glutathione peroxidase 4). In particular, this increase was particularly evident in the cortex and hippocampus of treated 3xTg-AD mice, which are parts of the brain that are compromised in AD [11]. In addition, the authors reported a decrease in Aβ and tau accumulation due to a prolonged treatment with mini-GAGR. Overall, the reduction in the oxidative stress, the neuroinflammation, and AD hallmarks, such as Aβ and tau, ameliorated the cognitive impairment of treated 3xTg-AD mice uniquely caused by a stimulation of Nr2 [11,28]. Like mini-GAGR, Gracilins (Figure 4d), the secondary metabolites derived from *Spongionella* sp., were proven to be Nrf2-stimulating compounds [78]. Leirós M et al. demonstrated that Gracilins are able to induce the Nrf2 translocation by inhibiting BACE1. After treatment, the authors registered, by an in vitro assay, increased levels of glutathione. Subsequently, by in vivo studies, the authors demonstrated the ability of Gracilins to reduce Aβ-42 and tau accumulation, with a positive trend on spatial memory and learning of treated mice [28,108].

### 6.4. Forsythoside A

In AD, iron deposits were observed in plaques, tangle-bearing neurons, and microglia [108]. Moreover, increasing data linked this deposition to AD progression [109,110,111].

Besides the toxicity of the iron accumulation itself, another iron neurotoxic mechanism can be attributed to the tendency for the undergoing of redox cycling between Fe^2+^ and Fe^3+^, producing ROS and increasing the oxidative stress condition, thus favoring tissue damage and cell death. This mechanism is known as ferroptosis [112]. For its marked presence in AD, ferroptosis becomes a good candidate for AD treatment [113]. Several studies have suggested that almost all genes related to ferroptosis are modulated by Nrf2 [114,115].

Forsythoside A (FA) (Figure 4e) treatment is known for its anti-AD properties exerted by the modulation of ferroptosis and inflammation targeting the activation of the Nrf2/GPX4 axis [116]. It is the principal active ingredient in the Chinese medicine *Forsythia suspensa*, with anti-inflammatory, antioxidant, antiviral, immunoregulatory, and neuroprotective properties [117]. Wang C. et al. demonstrated the protective role of Nrf2 signaling against ferroptosis in dopaminergic neurons. In FA-treated APP/PS1 mice, the authors registered a reduced Fyn phosphorylation and an increased expression level of GPX4, NAD(P)H quinone dehydrogenase 1 (NQO1), and Nrf2-mediated glycogen synthase kinase-3β (GSK-3β). Additionally, the authors observed an attenuation of inflammation by suppressing the activation of NF-kB signaling, which further modulated the expression of IL inflammation proneness and TNF-α [117,118].

As demonstrated by previous genetic and pharmacological studies, the attenuation of inflammation is ascribable to the Nrf2 activity, to the point that at the downregulation of Nrf2, it corresponds to an upregulation of expression levels of NF-kB [119]. The importance of the modulation of Nrf2 in AD also derives from ascertaining a sensible reduction in Aβ deposition in concomitance with the FA treatment in several cell lines and APP/PS1 mice [114].

### 6.5. Flavonoids

**Figure 5:**
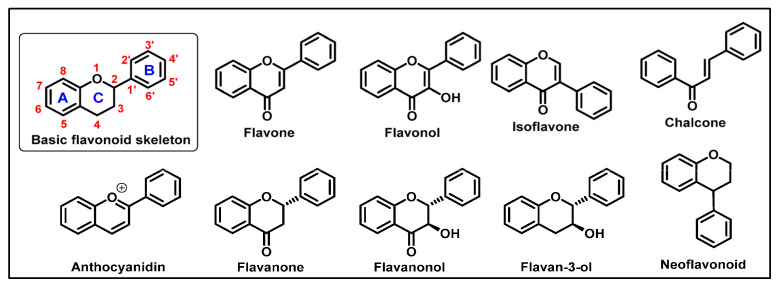
Chemical structure of flavonoids.

Flavonoids (Figure 5) are phytochemical polyphenolic compounds widely distributed in many plants, vegetables, leaves, and fruits. They possess several medicinal benefits, including antioxidant, antiviral, anti-inflammatory, and anticancer effects [120]. Chemically, flavonoids are constituted by a 15-carbon skeleton, which consists of two aromatic rings (A and B rings) and a heterocyclic ring (C ring) particularly embedded with oxygen atoms [121]. With a similar molecular mechanism of polyphenols, flavonoids are able to modulate the Nrf2 activity [121]. By in vitro studies, Li YR et al. examined the mechanism underlying the stimulation of Nrf2 activity by some flavonoids. Treating the cells with 7-O-methylbiochanin A (7-MBA), the authors registered an enhancement of Nrf2 translocation into the nucleus and a consequent upregulation of the Nrf2-dependent protein, including NQO1, and a key enzyme for GSH synthesis: γ-GCS (γ-glutamylcysteine synthetase). Additionally, the authors observed an inhibition of Nrf2 ubiquitylation and a modulation of Nrf2 phosphorylation by PI3K, MAPK, PKC, and PERK [122].

Actually, twenty-four compounds are Nrf2 inductors [123,124,125]. Dong et al. evaluated the effects of quercetin of neuronal apoptosis and cognitive abilities in d-galactose-induced neurotoxicity in mice. Via the activation of the Nrf2-ARE signaling pathway, they registered an induction of HO-1 expression and a modulation of Nrf2 mediating by p38-MAPK, contributing to contain the neurotoxicity effects induced by D-galactose, and neuronal cell apoptosis. Overall, the authors noticed that the stimulation of Nrf2 signaling, which is quercetin-dependent, improved the behavioral function in the treated mice [126]. For AD, several poly-herbal drug formulations were tested. The gingko leaf extract, particularly rich in flavonoids, was examined, in association with curcumin, in a phase 2 clinical trial against AD. It was demonstrated that the combination of these compounds significantly increased levels of GSH and decreased lipid peroxidation, when compared with the curcumin alone. This is likely attributable to the ability of the ginkgo extract to improve BBB permeability of curcumin, which improves neurological conditions in AD, including cognitive impairment [127].

## 7. Conclusions

Several studies have highlighted that polyphenolic compounds have an excellent anti-inflammatory and antioxidant activity that may prevent and modulate neurodegeneration. The majority of in vivo and in vitro studies attribute these activities to the ability of stimulating the antioxidant defense system by activating the Nrf2 pathway, which is badly damaged in AD patients. Therefore, the activation of Nrf2 signaling by employing anti-inflammatory diet patterns may reinforce the antioxidant defense system, enhancing the cellular response to the oxidative attacks. As we strongly believe that this is a possible route to take, in our ongoing study, we are analyzing whether a high phenolic-rich food intake can attenuate neuroinflammation associated with AD, by monitoring the neuroinflammatory landscape in AD patients and the role of Nrf2 signaling in response to a personalized anti-inflammatory diet. This review and our ongoing study may be an opportunity to encourage the treatment of AD patients with a holistic approach, and to promote the development of new potential Nrf2-targeting drugs.

## Figures and Tables

**Table 1:** The principal clinical trials involving Nrf2 in AD. Data from ClinicalTrials.gov (accessed on 9 August 2024).

Number Identifier	Study Type	Compound	Data/Status	Outcomes	Main Side Effects
NCT02711683 [74]	Observational	DL-3-*n*-butylphthalide	2019-12 (completed)	Slower cognitive decline	Gastrointestinal disorders, including nausea, vomiting, and gastrointestinal discomfort Abnormal liver function Neurological disorders, including insomnia, dizziness, fatigue, and psychiatric symptoms [75]
NCT02292238 [76]	Interventional	Benfotiamine	2022-06 (completed)	Reduced AD-like changes in tangles, and plaques. Diminished inflammation, neuron loss, and memory	Data not provided [77]
NCT015004854		Resveratrol	2014-03 (completed)	Modest improvement of cognitive function	Data not provided
NCT00164749 [78]		Curcumin	2006-07 (completed)	No differences in Aβ levels between treatments or MMSE scores	Gastrointestinal discomfort, chest tightness, skin rashes, and swollen skin, and allergic reactions, including dermatitis [79]
NCT04213391 [80,81]		Sulforaphane	2022-12 (completed)	Results awaited	Brain swelling, microbleeds, fatigue, nausea [82]
NCT01058941 [83]		Alpha Lipoic Acid and Omega-3 Fatty Acid	2014-12 (completed) [60]	Greater impairment in functional and cognitive ability	Cardiac disorders, including tachycardia and atrial fibrillations. Gastrointestinal disorders, including gastrointestinal obstruction, gastric ulcer, and hemorrhage. Pneumonia. Head injury. Pleura effusion, subdural hematoma evacuation. Fatigue, allergic conditions. Behavioral and psychiatric symptoms of dementia
NCT02085265 [84]		Perindopril	2023-09 (recruiting) [61]	Results not provided	Data not provided
NCT00439166 [85]		Doxycycline	2010-12 (completed)	Results not provided	Data not provided
NCT04063124 [86]		Quercetin	2023-01 (completed)	Safety, tolerability, and feasibility of the treatment	Diarrhea and emesis, urinary tract infection, hypoglycemia
NCT01982578 [87]		Genistein	2020-12 (completed) [62]	Lower Aβ deposition and slowdown of cognitive decline in prodromal AD patients	Mild diarrhea
NCT00948259 [88]		Tideglusib	2009-30 (completed) [63]	Safety of the drug. Positive, but not significant, trends in cognitive health	Data not provided
NCT00056225 [89]		Pyridoxine	2009-30 (completed)	Results not provided	Depression
NCT03289143 [90]		Semorinemab	2021-01 (completed)	Well-tolerated and acceptable safety profile No slowdown of AD progression	Nasopharyngitis, and infusion-related reaction
